# Missense Mutations Allow a Sequence-Blind Mutant of SpoIIIE to Successfully Translocate Chromosomes during Sporulation

**DOI:** 10.1371/journal.pone.0148365

**Published:** 2016-02-05

**Authors:** Baundauna Bose, Sydney E. Reed, Marina Besprozvannaya, Briana M. Burton

**Affiliations:** Department of Molecular and Cellular Biology, Harvard University, Cambridge, Massachusetts, United States of America; ContraFect Corporation, UNITED STATES

## Abstract

SpoIIIE directionally pumps DNA across membranes during *Bacillus subtilis* sporulation and vegetative growth. The sequence-reading domain (γ domain) is required for directional DNA transport, and its deletion severely impairs sporulation. We selected suppressors of the *spoIIIEΔγ* sporulation defect. Unexpectedly, many suppressors were intragenic missense mutants, and some restore sporulation to near-wild-type levels. The mutant proteins are likely not more abundant, faster at translocating DNA, or sequence-sensitive, and rescue does not involve the SpoIIIE homolog SftA. Some mutants behave differently when co-expressed with *spoIIIEΔγ*, consistent with the idea that some, but not all, variants may form mixed oligomers. In full-length *spoIIIE*, these mutations do not affect sporulation, and yet the corresponding residues are rarely found in other SpoIIIE/FtsK family members. The suppressors do not rescue chromosome translocation defects during vegetative growth, indicating that the role of the γ domain cannot be fully replaced by these mutations. We present two models consistent with our findings: that the suppressors commit to transport in one arbitrarily-determined direction or delay spore development. It is surprising that missense mutations somehow rescue loss of an entire domain with a complex function, and this raises new questions about the mechanism by which SpoIIIE pumps DNA and the roles SpoIIIE plays *in vivo*.

## Introduction

Impairing a protein and looking for ways in which its function can be restored can lead to new discoveries about how the protein works. This type of study often reveals new insight about the mechanism by which the protein acts or the role the protein plays *in vivo*. The protein in this study is a DNA transporter (SpoIIIE) that employs a specific domain (γ) to read specific sequences and transport DNA directionally. Here we report that, in the absence of that dedicated DNA-reading domain, missense mutations elsewhere in the protein somehow compensate for a defect which normally results from sequence insensitivity. These simple mutations surprisingly rescue the loss of a complex domain function, and thus uncover new questions about the role SpoIIIE plays in sporulation, a process for which it is essential.

SpoIIIE is required for sporulation, a developmental process that allows populations of *B*. *subtilis* to survive adverse conditions by transitioning to a highly protected form [1–4]. During this process, an asymmetric septum divides a larger compartment (mother cell) and a smaller compartment (forespore). The mother cell membranes then migrate around the forespore engulfing it. The forespore develops a protective coat and transitions to a dormant state. Finally, the mother cell lyses to release the mature spore.

As cells sporulate, the chromosomal DNA undergoes a distinctive series of movements. At the onset of sporulation, two circular chromosomes are present in each cell. These chromosomes are remodeled into an “axial filament”, with the origin of each chromosome tethered to opposite cell poles and the termini near midcell [5–7]. When the asymmetric septum forms, it traps ~25–30% of one chromosome in the forespore [1, 6, 8, 9]. The remaining portion of this chromosome is then pumped into the forespore by the DNA translocase SpoIIIE [1, 10, 11].

SpoIIIE is a powerful and processive DNA transporter during sporulation. This transporter moves ~70–75% of the chromosome (3Mb) into the forespore in 20 minutes [12]. SpoIIIE also maintains separation of the mother cell from the forespore, stripping proteins from the DNA during transport and thus enabling specialized programs of gene expression to proceed in each cellular compartment [2, 13–17]. SpoIIIE is required for membrane fission when mother and forespore membranes separate during asymmetric septation and is involved in membrane fission at the completion of spore engulfment [12, 14, 18, 19, 20].

SpoIIIE is a member of the SpoIIIE/FtsK family of proteins, which are conserved in most bacteria [21]. Members of this family function in chromosome transport, transfer of conjugative DNA, and protein export [22–24]. SpoIIIE/FtsK family members form hexameric rings [25–27]. Each protein typically has an N-terminal transmembrane domain, a linker domain, a motor domain composed of 2 separate subdomains (α and β), and a γ domain [25, 28]. The N-terminal domain of SpoIIIE localizes it to the septum and later promotes membrane fission [14, 15, 20, 29, 30]. The linker varies substantially between family members and is thought to be unstructured. The α subdomain is unique to the SpoIIIE/FtsK family of RecA-like ATPases, whereas the β subdomain is more widespread and has canonical Walker A and B motifs for ATP binding and hydrolysis respectively [21]. The γ domain senses specific DNA sequences and confers directionality to transport [31–35]. For the γ domain of *B*. *subtilis* SpoIIIE, modulation of DNA translocation and specific sequence recognition are functions that are supported by published findings; however other roles for this domain are possible based on the functions of homologous proteins and as hypothesized by others [30].

The SpoIIIE γ domain detects 8-nucleotide motifs called SRSs (SpoIIIE-Recognition Sequences) to determine directionality of DNA transport [33]. These types of sequences are highly conserved, and their orientation on the *B*. *subtilis* chromosome is extremely biased; 82% are found on the leading strand [33, 36, 37]. The γ domain regulates ATPase activity of the motor domain in response to SRSs [26, 38]. Strains harboring *spoIIIE* alleles that lack the gamma domain (*spoIIIEΔγ)* exhibit a 4- to 5- order-of-magnitude decrease in sporulation efficiency. These strains frequently pause and reverse chromosome transport during sporulation [33].

Here, we report that the sporulation defect of *spoIIIEΔγ* cells can be partly or fully rescued by any of a variety of missense mutations in the linker and motor domains of SpoIIIEΔγ. The mutations rescue chromosome transport during sporulation, but not to wild-type levels. The *spoIIIE* homolog *sftA* is not required for rescue. The suppressor mutations do not alter protein levels *in vivo* or ATPase activity *in vitro*. Strains expressing both *spoIIIEΔγ* and intragenic suppressor alleles sporulate to varying extents, consistent with the idea that some variants may form mixed oligomers and others may not. The missense mutations in full-length *spoIIIE* have no effect on sporulation, and yet the corresponding residues are rarely found in other SpoIIIE/FtsK family members.

The finding that missense mutations elsewhere in the protein somehow compensate for loss of the entire domain responsible for DNA sequence recognition and orienting transport is surprising. However, we also found that the suppressors do not rescue chromosome translocation defects during vegetative growth, indicating that the role of the γ domain cannot be fully replaced by these mutations. We propose two models for how the suppressors might function that are consistent with these observations and that highlight how the mutants could be used to further explore and understand the role of SpoIIIE in sporulation.

## Materials and Methods

### Media and growth conditions

Except where otherwise specified, *B*. *subtilis* and *E*. *coli* cells were grown at 37°C in liquid LB with aeration or on LB agar plates. When appropriate, antibiotics were used at the following concentrations: chloramphenicol (5μg ml^-1^), kanamycin (5μg ml^-1^) to select kan or neo resistance, tetracycline (10μg ml^-1^), spectinomycin (100μg ml^-1^), erythromycin (0.5μg ml^-1^) and lincomycin (12.5μg ml^-1^) together to select for macrolide-lincosamide-streptogramin B (mls) resistance, and ampicillin (100μg ml^-1^). Because of gene conversions that restored *spoIIIE* at the native locus observed during suppressor selection, strains bearing *ΔspoIIIE* alleles were always streaked on plates that select for the *ΔspoIIIE* marker at the outset of each experiment.

For sporulation by exhaustion, *B*. *subtilis* cells were grown in at 37°C DSM broth for 24–36 hrs. DSM broth was made as previously described [39], except containing 1mM MgS0_4_. To eliminate unsporulated cells and select spores, cultures were heat-killed at 80°C for >20min. To determine sporulation efficiencies, heat-killed cultures diluted in T Base [39] plus 1mM MgS0_4_ and plated onto DSM plates to measure colony forming units (cfu). Sporulation by resuspension was performed as previously described [39], except that CHI+II at pH 6.1 and not pH 7 was used.

### Strains and alleles

Strain and plasmid construction are described in the supporting information. Table A in S1 Text lists *B*. *subtilis* strains and Table B in S1 Text lists primers used in this study.

### Suppressor selection and identification

Intragenic suppressors were selected by subjecting cells to repeated rounds of sporulation by exhaustion and heat kills. Each culture that was passaged in this way is referred to as a “perpetuated culture”. Cultures were initially inoculated with cells from streaks from freezer stocks, and after that new cultures were inoculated by 10^−1^ dilution of the previous heat-killed culture into fresh DSM broth. Heat-killed cultures were periodically plated for cfu. When this plating indicated a significant increase in the sporulation efficiency, individual colonies were selected and streaked to single colonies ≥3 times. For each single isolate, antibiotic resistance was verified by patching, and the efficiency of sporulation by exhaustion was evaluated. In several cases, isolates lost the marker indicating *ΔspoIIIE*, and recovered wild-type sporulation efficiency. These isolates were not studied further. From each perpetuated culture, the *spoIIIEΔγ* sequence for one or two single isolates was determined. Genomic DNA was obtained from the isolate, *spoIIIEΔγ* at *ycgO* was amplified by PCR with Phusion (Finnzymes or New England Biolabs), and the PCR was sequenced (Genewiz).

Initially, bBB388 was used for the selection. Because P492Q was obtained several times, the likelihood of obtaining this mutation again was reduced by mutating the codon for P492 from *ccg* to *cct*. In the resulting allele, two substitutions rather than one would be required to obtain P492Q. Because *ΔspoIIIE*::*spc* frequently underwent gene conversion to restore *spoIIIE* at the native locus, *ΔspoIIIE*::*neo* was used instead. This resulted in BOSE2282, which was used for the next batch of perpetuated cultures. The *ΔspoIIIE*::*neo* allele in BOSE2282 underwent gene conversion to restore *spoIIIE* at the native locus even more frequently than *ΔspoIIIE*::*spc*, so strains with the mutated P492 codon and either *ΔspoIIIE*::*spc* (BOSE2425) or *ΔspoIIIE*::*mls* (BOSE2427) were used for suppressor selection thereafter. The extents of various *spoIIIE* deletions and the sequence present in ectopic *spoIIIEΔγ* constructs are illustrated in S1 Fig.

### Microscopy

Cells were induced to sporulate by resuspension. At indicated timepoints, samples were centrifuged at 6K x g for 1.5 min., resuspended in 1x PBS plus 9.5μM FM4-64 (Life Technologies), and imaged by fluorescent microscopy as described previously [12]. Image analysis was performed using ImageJ [40].

### Western blots

Cells were induced to sporulate by resuspension. Vegetative samples were taken from cultures in CH medium prior to resuspension, and sporulating samples were taken 2.5 hours after resuspension. Cells were pelleted at 21.1K rcf for 3 min., and pellets were stored at -80°C. Pellets were thawed, resuspended in a volume of lysis buffer (20mM Tris pH7.5, 10mM EDTA pH8.0, 1mg ml^-1^ lysozyme, 1mM PMSF) proportional to the OD600 of the original sample, and incubated at 37°C for 10 min. SDS loading buffer was added. Each sample was incubated at 50°C for 10 min., vortexed briefly, and pelleted at 21.1K rcf for 1 min.

Proteins were separated by SDS-PAGE on 7% Tris-Glycine gels and transferred to Whatman PDF (GE Healthcare) using the Trans-blot semi-dry transfer apparatus (Bio-Rad) at 10V for 60 min. Membranes were blocked in TBST (10mM Tris, 150mM NaCl, 0.05% Tween-20, pH 8) plus 5% milk at 4°C for 30 min, incubated in 1:10,000 affinity purified anti-SpoIIIE polyclonal antisera in TBST plus 5% milk at 4°C overnight, washed several times in TBST, incubated in 1:10,000 donkey anti-rabbit HRP (Jackson ImmunoResearch) in TBST at room temp. for 1 hr 15 min., and washed several times in TBST. Anti-SpoIIIE polyclonal antisera (custom order, Cocalico Biologicals) were affinity purified against wild-type, soluble SpoIIIE using Affigel-15 (Bio-Rad). Signals were detected using Western lightning *Plus*-ECL reagents (Perkin-Elmer) and a ChemiDoc XRS Imaging System (Bio-Rad). Quantification of bands on Western blots was performed using Quantity One (Bio-Rad).

### Protein purification and ATPase assays

Soluble variants of SpoIIIE were expressed, purified, and stored as described previously [38]. Soluble wild-type SpoIIIE was expressed from pJB103 [11]. Soluble SpoIIIEΔγ variants were expressed from pMB041 (SpoIIIEΔγ), pBOSE2155 (SpoIIIEΔγ(Y316D)), pBOSE2159 (SpoIIIEΔγ(A343V)), and pBOSE2153 (SpoIIIEΔγ(P492Q)).

ATPase activity was measured using an NADH+-coupled assay as previously described [11, 38, 41]. Where indicated, PY79 genomic DNA was added to a final concentration of 13 ng μl^-1^, or 20 μM base pairs.

### Comparative sequence analysis

Two groups of SpoIIIE/FtsK family members were analyzed. One group had γ domains while the other did not. The group containing a γ domain consisted of the 84 proteins in the Pfam DUF4117 seed alignment that also had FtsK_SpoIIIE and Ftsk_gamma domains [42]. The group without a γ domain consisted of the 47 proteins annotated in Pfam as having DUF4117 and FtsK_SpoIIIE domains, but no Ftsk_gamma domains. Each group of proteins was aligned with *B*. *subtilis* SpoIIIEΔγ using the ClustalW [43] function in Geneious (Biomatters). The similarity between γ domains of SpoIIIE (residues 716–782) and SftA (residues 881–947) was also measured following alignment using the ClustalW function in Geneious.

### Statistical analysis

Statistical analysis was performed using the Analysis ToolPak in Microsoft Excel. Single factor ANOVA testing was performed to determine whether a group of means was significantly different from each other. Pairwise comparisons were assessed using t-tests. For each pair an F-test was first run to determine whether variances were equal or unequal. Based on the results of the F-tests, t-tests assuming equal or unequal variances, as appropriate, were run.

## Results

### Selection and identification of suppressors that rescue the sporulation defect of *spoIIIEΔγ* cells

To learn more about the importance of sequence recognition during DNA transport in sporulation, and about the role of the SpoIIIE γ domain in sporulating cells, we selected strains that coped better with the absence of the SpoIIIE γ domain. To select for mutants that improved the low sporulation efficiency of *spoIIIEΔγ* cells, we subjected *spoIIIEΔγ* cultures to successive rounds of sporulation and outgrowth. Each “*spoIIIEΔγ*” strain harbored an ectopic construct with *spoIIIEΔγ* under control of the native *spoIIIE* promoter and a marked deletion of *spoIIIE* at the native locus (Table A in S1 Text, Table C in S1 Text, S1 Fig). In each round, cultures were induced to sporulate in DSM, cultures were incubated at high temperature to select spores and eliminate cells, and the heat-killed culture was used to inoculate fresh DSM for the next round. Sporulation efficiency was evaluated periodically by dilution and plating of the heat-killed culture. Single isolates were obtained from cultures that exhibited elevated sporulation efficiencies, and the sporulation efficiency of each single isolate was assayed. For one or two single isolates per perpetuated culture, the *spoIIIEΔγ* construct was sequenced (Table C in S1 Text).

Many of the suppressor isolates harbored mutations within *spoIIIEΔγ*. Others did not and were classified as extragenic suppressors. These isolates were preserved for future analysis, and will not be discussed further here. Some perpetuated cultures underwent gene conversion to restore *spoIIIE* at the native locus during selection. The *ΔspoIIIE* marker was lost, and the isolates sporulated at wild-type levels. This presumably resulted from repair between the ectopic *spoIIIEΔγ* allele and *spoIIIE* deletion alleles that only deleted part of the *spoIIIE* sequence, leaving the portion encoding the γ domain intact. The extent of each deletion in *spoIIIE* and its overlap with the ectopic *spoIIIEΔγ* sequence is shown in S1 Fig.

### Intragenic suppressor mutations were found throughout the linker and motor domains

**Fig 1:**
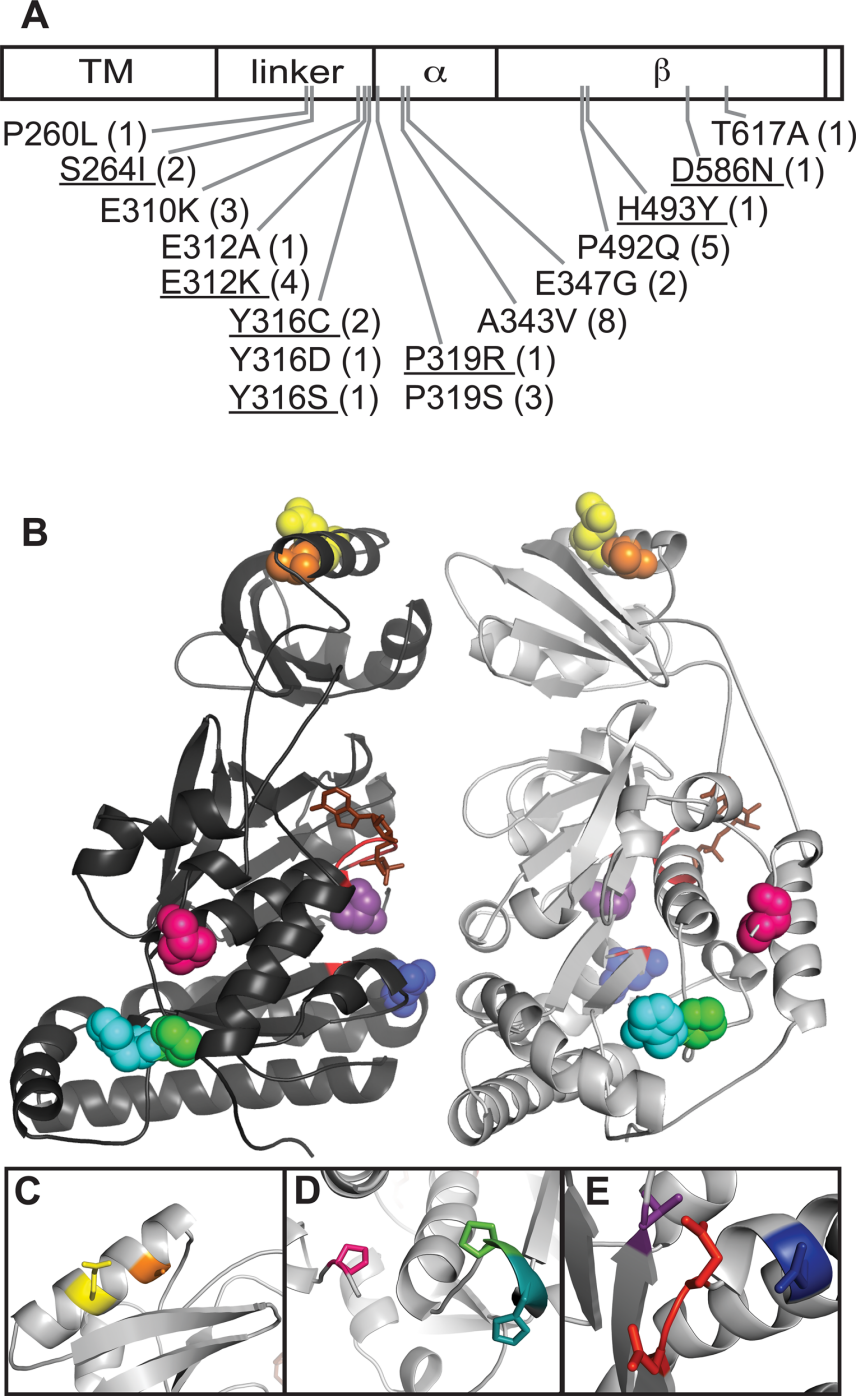
Intragenic *spoIIIEΔγ* suppressor mutations alter residues in the linker and motor domains. A. Positions of intragenic suppressor mutations are indicated on a schematic of the *spoIIIEΔγ* linear sequence. The γ domain is not shown, but is C-terminal to the β subdomain in full-length SpoIIIE. Numbers in parentheses indicate the number of times each mutation was isolated. Underlined mutations were identified only after the P492 codon was mutated from ccg to cct to lessen the chances of obtaining P492Q. In this study, SpoIIIE codons are numbered for the protein beginning with the sequence MSVAKKKRKS. This presumes an earlier translation start and thus the codons are numbered +2 relative to some annotations of SpoIIIE [50]. (B-E). Positions of intragenic suppressor mutations are shown in a 3D-model of the SpoIIIE motor domain, obtained by threading the SpoIIIE sequence onto a FtsK crystal structure [25, 44]. B. Two of the six subunits of a SpoIIIE hexamer are shown. The Walker A and Walker B sites are shown in red and ADP is shown in brown. P319 (pink), A343 (orange), E347 (yellow), P492 (green), H493 (cyan), D586 (blue), and T617 (purple) are shown as space-filled residues. C. A343 (orange) and E347 (yellow) lie on the same face of a helix in the α domain. D. P319 (pink), P492 (green) and H493 (cyan) are near each other. E. D586 (blue) and T617 (purple) are near the Walker B motif (red) in the β domain.

The intragenic suppressor mutations are shown in Fig 1A. Among the 37 independent intragenic suppressor isolates, 16 unique mutations were found at 12 different residues. Because P492Q was recovered several times from the first few sets of cultures, we reduced the likelihood of obtaining this mutation by mutating the P492 codon from ccg to cct. At least two mutations would be required to change the cct codon for Proline to one encoding Glutamine. P492Q was not isolated from subsequent selection rounds with the mutated strain.

Intragenic suppressor mutations were found throughout the soluble portions of *spoIIIEΔγ* (Fig 1A). Positions of mutated residues are shown in an approximation of the SpoIIIE motor domain structure, made by threading the SpoIIIE sequence onto an FtsK structure (Fig 1B) [25, 44]. While no clear overall pattern emerged from this structural model, some sets of mutated residues are clustered together. Two mutated residues (A343 and E347) appear to lie on the same face of a helix in the α domain (Fig 1C), three (P319, P492, and H493) appear to be in close proximity in the β domain (Fig 1D), and two (D586 and T617) are somewhat near the Walker B motif (Fig 1E). Many mutated residues are in the linker domain and are thus cannot be mapped onto any currently available structure.

### Single amino acid changes in SpoIIIEΔγ can rescue sporulation

**Fig 2:**
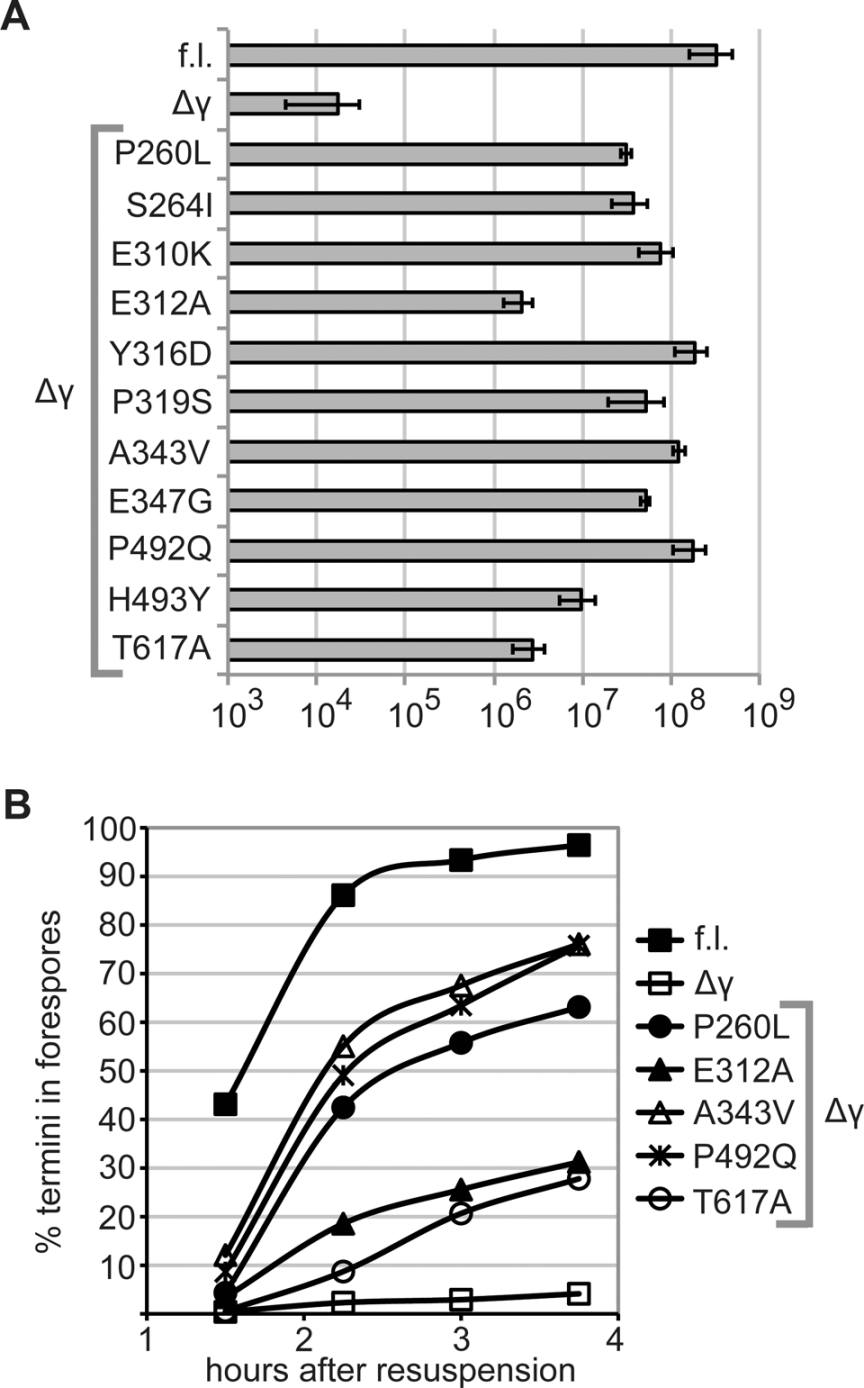
Missense mutations in *spoIIIEΔγ* rescue sporulation and chromosome transport *in vivo*. A. Suppressor mutations rescue spore formation. Intragenic mutations identified by suppressor selection were remade in a *spoIIIEΔγ* allele by site-directed mutagenesis and expressed from the *spoIIIE* promoter at an ectopic locus (*ycgO*) in a *ΔspoIIIE* strain. Strains were induced to sporulate for 24–36 h in DSM medium, unsporulated cells were eliminated by heat-kill, and the number of spores was measured by plating for cfu. The number of heat-resistant spores per ml is indicated for strains harboring full-length (“f.l.”) *spoIIIE* (bKM776), *spoIIIEΔγ* (BOSE2042), and 11 *spoIIIEΔγ* mutants: P260L (BOSE2286), S264I (BOSE2540), E310K (BOSE2411), E312A (BOSE2121), Y316D (BOSE2284), P319S (BOSE2321), A343V (BOSE2288), E347G (BOSE2323), P492Q (BOSE2120), H493Y (BOSE2538), and T617A (BOSE2123). Each number is the average of at least 3 replicates. Error bars indicate one standard deviation in each direction. B. Suppressor mutations rescue chromosome transport *in vivo*. Sporulation was induced by resuspension and DNA transport was evaluated using a previously-established fluorescent microscopy assay [12]. *yfp* and *cfp* genes are expressed from a forespore-specific promoter (*PspoIIQ*). *yfp* is integrated near the origin (*yycR*), and its expression indicates that asymmetric septation is complete. *cfp* is integrated near the terminus (*pelB*), and its expression indicates that the terminus has been transported into the forespore. Percent of termini in forespores is the percent of YFP+ cells that are also CFP+. Data are shown for full-length (“f.l.”) *spoIIIE* (bBB128), *spoIIIEΔγ* (bBB412), and 5 *spoIIIEΔγ* mutants: P260L (BOSE2331), E312A (BOSE2201), A343V (BOSE2332), P492Q (BOSE2200), and T617A (BOSE2202). Each data point represents the average of ≥ 3 replicates, with ≥ 500 forespores scored for each.

We found the missense mutations in SpoIIIEΔγ were responsible for rescue of sporulation. Point mutations in *spoIIIEΔγ* from several suppressor isolates were remade in the *spoIIIEΔγ* construct by site-directed mutagenesis of plasmids that were cloned in *E*. *coli*, sequenced, and used to transform *B*. *subtilis*. Integration of ectopic constructs by double crossover was verified, and strains including the resulting constructs were assayed for sporulation by exhaustion (Fig 2A). All tested mutants rescued sporulation comparably to the original isolates from the selection, demonstrating that in each case the phenotype was caused by mutation of a single residue.

Rescue of the sporulation defect by the suppressor mutants involves improved translocation of the chromosome. We tested the effect of five intragenic suppressor mutants on chromosome translocation during sporulation using a previously described DNA transport assay (Fig 2B)[12]. This assay employs strains with two fluorescent reporters expressed from the same early, forespore-specific promoter (*PspoIIQ*). *PspoIIQ-yfp* is integrated in the chromosome near the origin, and expression of *yfp* indicates cells that have completed asymmetric septation. *PspoIIQ-cfp* is integrated near the terminus, and expression of *cfp* indicates cells that have successfully translocated the terminus into the forespore. The ratio of CFP+ to YFP+ cells reflects the fraction of forespores that have received entire chromosomes. Successful transport of the terminus could result from either faster translocation in individual cells or from stabilization of sporulating cells against lysis. If cells that normally lyse are stabilized and remain intact longer, they are more likely to receive the terminus even if the terminus is not translocated into the forespore faster. Thus the output from this assay does not strictly indicate the translocation rate. The five tested suppressor mutants were more likely than *spoIIIEΔγ* cells to have completed chromosome transport at each assayed timepoint (Fig 2B). There is a rough correlation between successful chromosome transport and sporulation (Fig 2). Mutants that were better at chromosome transport (P492Q and A343V) also exhibited the strongest rescue of sporulation. Mutants that transported chromosomes less well (E312A and T617A) also exhibited a more modest rescue of sporulation.

### SftA is not required for rescue

**Fig 3:**
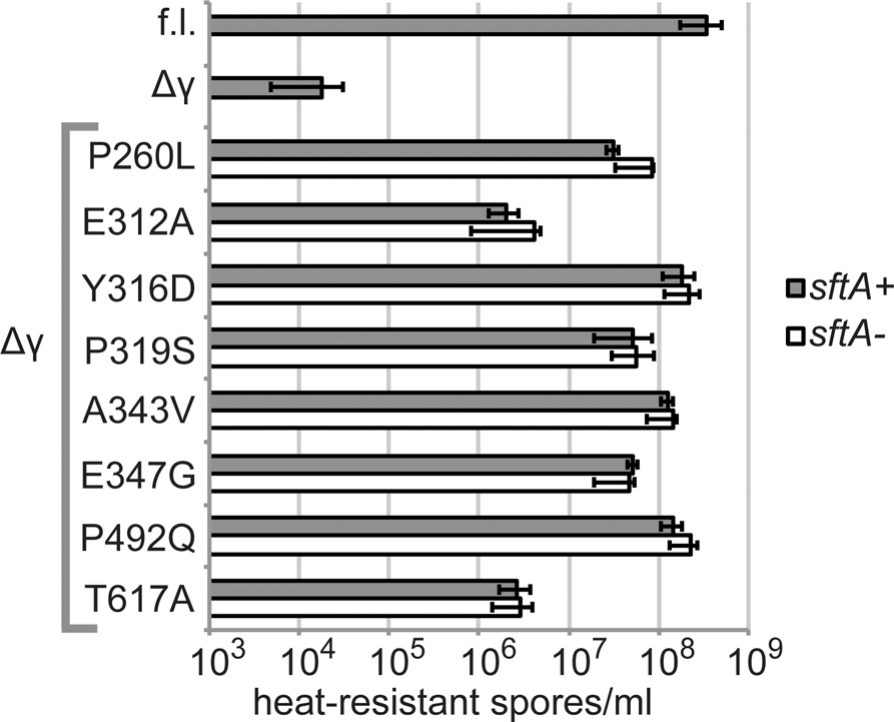
SftA is not required for rescue of spore formation by *spoIIIEΔγ* intragenic suppressor mutations. Strains were induced to sporulate for 24–36 h in DSM medium, unsporulated cells were eliminated by heat-kill, and the number of spores was measured by plating for cfu. Sporulation levels are similar for *sftA+* and Δ*sftA* strains bearing *spoIIIEΔγ* mutant alleles: P260L (BOSE2286; BOSE2492), E312A (BOSE2121; BOSE2486), Y316D (BOSE2284; BOSE2490), P319S (BOSE2321; BOSE2496), A343V (BOSE2288; BOSE2494), E347G (BOSE2323; BOSE2498), P492Q (BOSE2120; BOSE2935), and T617A (BOSE2123; BOSE2488). Sporulation efficiencies for strains harboring full-length (“f.l.”) *spoIIIE* (bKM776) and *spoIIIEΔγ* (BOSE2042) are plotted for comparison. The average of at least 3 replicates is plotted. Error bars indicate one standard deviation in each direction.

We investigated whether SpoIIIEΔγ suppressor mutants might sporulate better due to an effect of the SpoIIIE homolog SftA, and we found that SftA is not required for rescue. SftA localizes to nascent division sites and promotes accurate chromosome partitioning [45]. Unlike SpoIIIE, SftA does not appear to have a transmembrane domain, and it cannot rescue DNA that is trapped in a division septum. However, SftA does have a γ domain. It is not yet known whether SftA specifically recognizes the same sequences (SRSs) as SpoIIIE. However SftA is likely to recognize similar if not identical sequences, since both proteins transport the same chromosome directionally, both proteins have similar γ domains (55.2% identical, 68.7% positive in BLOSUM62 alignment), and even the sequences recognized by *Escherichia coli* FtsK (KOPS) are extremely similar to those recognized by SpoIIIE [35]. In fact, replacing the γ domain of SpoIIIE with that of FtsK in *B*. *subtilis* supported near-wild-type sporulation levels [33]. Given the related roles of SftA and SpoIIIE in DNA transport, and the likelihood that they recognize similar sequences, we hypothesized that SftA might play a role in the rescue of *spoIIIEΔγ* strains. For example, the SpoIIIEΔγ mutants could have formed mixed oligomers with SftA that used the SftA γ domain to read specific DNA sequences and transport DNA directionally. However, we found that rescue of sporulation in the intragenic suppressor mutants occurs independently of SftA, as rescue was unaltered in *sftA*-deleted strains (Fig 3).

### Mutations do not alter protein levels *in vivo* or ATPase activity *in vitro*

**Fig 4:**
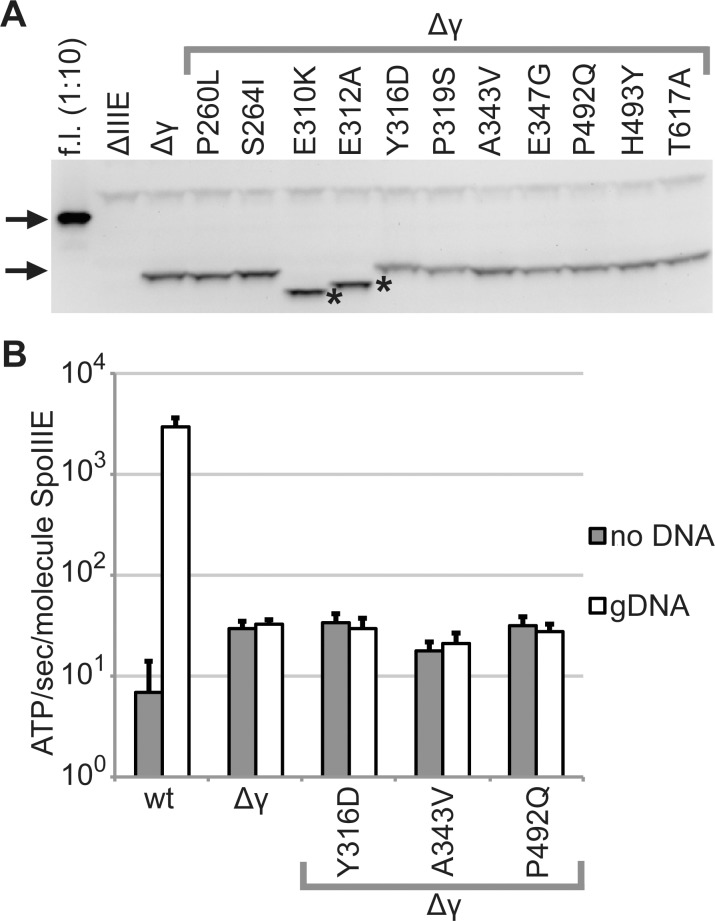
Intragenic suppressor mutations do not alter protein levels or ATPase activity. A. SpoIIIE levels are similar in strains expressing *spoIIIEΔγ* or a suppressor allele. Samples were harvested 2.5 h after cells were induced to sporulate by resuspension. Protein levels were evaluated using Western blots with antibodies against SpoIIIE. The upper arrow indicates the position of full-length SpoIIIE, and the lower arrow indicates the position of SpoIIIEΔγ on the blot. Asterisks indicate SpoIIIEΔγ variants whose migration was altered. All strains bear Δ*spoIIIE*::*neo*. As indicated, samples were from strains with full-length (“f.l.”) *spoIIIE* (1:10 dilution; bKM776), no ectopic *spoIIIE* (bDR1066), *spoIIIEΔγ* (BOSE2042), or a *spoIIIEΔγ* mutant: P260L (BOSE2286), S264I (BOSE2540), E310K (BOSE2411), E312A (BOSE2121), Y316D (BOSE2284), P319S (BOSE2321), A343V (BOSE2288), E347G (BOSE2323), P492Q (BOSE2120), H493Y (BOSE2538), T617A (BOSE2123). One representative set from at least three replicates is shown here. All three replicates are shown in S2B Fig. ATPase activity of intragenic suppressor mutants is similar to that of SpoIIIEΔγ. ATPase activity of soluble variants of SpoIIIE and SpoIIIEΔγ was measured using an NADH+-coupled assay, as described previously [11, 38, 41]. Results obtained in this study for these two proteins are similar to those obtained previously [38]. Three different intragenic suppressor mutations were introduced to the SpoIIIEΔγ-encoding plasmid by site-directed mutagenesis, and the resulting soluble versions of each mutant were expressed in and purified from *E*. *coli*. Each suppressor mutant exhibited the same ATPase activity as that of SpoIIIEΔγ. Averages of at least three replicates are plotted. Error bars indicate plus one standard deviation.

Mutations in a protein that increase its cellular abundance might also increase its activity *in vivo*. We found that protein levels of the suppressor mutants were similar to those of SpoIIIEΔγ during sporulation (Fig 4A and S2 Fig). We detected SpoIIIE protein levels in samples collected from *B*. *subtilis spoIIIE*, *spoIIIEΔγ*, and *spoIIIEΔγ* suppressor strains while cells were sporulating, using western blots with anti-SpoIIIE antibodies. Levels of full-length SpoIIIE appeared to be much higher than those of SpoIIIEΔγ, but this could result from the antibody having higher affinity for full-length SpoIIIE or from higher protein levels *in vivo*. Levels of all tested SpoIIIEΔγ variants appeared comparable to each other. Quantification revealed band intensities of SpoIIIEΔγ mutants were lower or less than two-fold higher than that of SpoIIIEΔγ, and there was no significant difference between mean intensities for each SpoIIIEΔγ variant by single factor ANOVA (p = 0.316). The E310K and E312A variants migrated faster in the gel than the other SpoIIIEΔγ variants, perhaps because even a slight change in charge in this region of SpoIIIEΔγ alters its properties or because these variants were proteolytically processed.

We hypothesized that the suppressor mutants might improve sporulation by making SpoIIIEΔγ a more efficient ATPase, perhaps by allowing some stimulation in the presence of DNA. We found that ATPase rates of three suppressor mutant proteins were indistinguishable from those of SpoIIIEΔγ and insensitive to the presence of genomic DNA. We purified soluble SpoIIIEΔγ variants from *E*. *coli*, and tested their ATPase activity *in vitro* (Fig 4B). In these assays, wild-type SpoIIIE exhibited a low basal ATPase rate that was greatly stimulated by genomic DNA. SpoIIIEΔγ exhibited an elevated basal ATPase rate compared with wild-type SpoIIIE, and the ATPase rate of SpoIIIEΔγ was not stimulated by genomic DNA. These trends for wild-type SpoIIIE and SpoIIIEΔγ were previously observed [38]. Three SpoIIIEΔγ suppressor mutants were tested (Y316D, A343V, and P492Q), and each one showed similar ATPase activity to that of SpoIIIEΔγ.

### Genetic characterization of mutants

**Fig 5:**
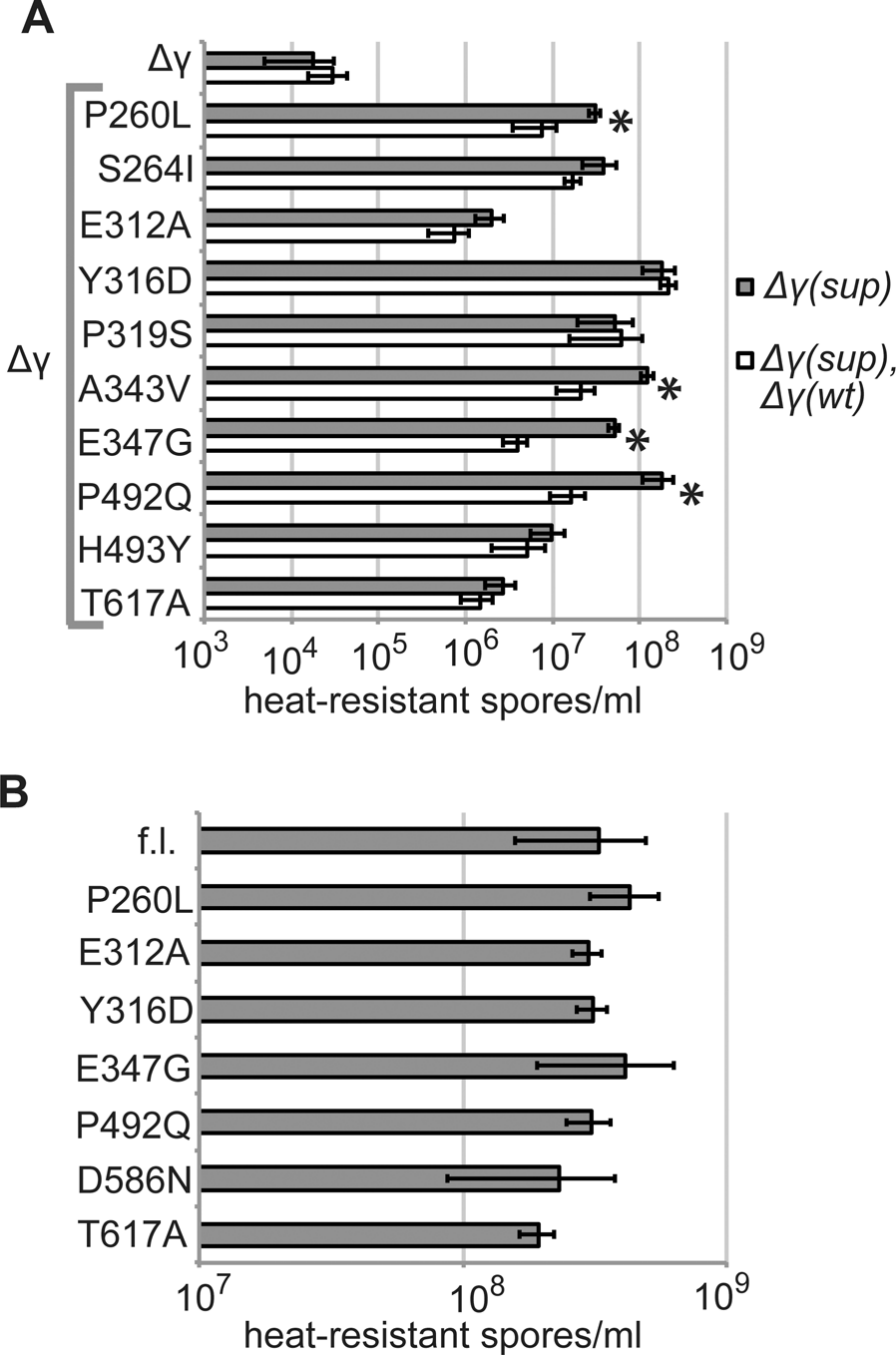
Spore formation in various genetic backgrounds. Strains were induced to sporulate for 24–36 h in DSM medium, unsporulated cells were eliminated by heat-kill, and the number of spores was measured by plating for cfu. The average of at least 3 replicates is plotted. Error bars indicate one standard deviation in each direction. A. Intragenic mutants are partially or fully dominant to *spoIIIEΔγ*. Strains expressing both *spoIIIEΔγ* and an intragenic *spoIIIEΔγ* suppressor mutant exhibit sporulation efficiencies that are intermediate between those of strains with only the *spoIIIEΔγ* or the mutant allele, or that are similar to that of the mutant allele. All displayed strains express the indicated *spoIIIE* allele from the *spoIIIE* promoter at the ectopic locus *ycgO*. White bars indicate strains that also express *spoIIIEΔγ* from the *spoIIIE* promoter at the ectopic locus *yhdGH*. Asterisks mark pairs of sporulation efficiencies that were significantly different from each other by t-tests (p<0.05). Sporulation efficiencies are shown for *spoIIIEΔγ* (BOSE2042; BOSE2301) and 10 *spoIIIEΔγ* mutants: P260L (BOSE2286; BOSE2311), S264I (BOSE2540; BOSE3089), E312A (BOSE2121; BOSE2305), Y316D (BOSE2284; BOSE2309), P319S (BOSE2321; BOSE3083), A343V (BOSE2288; BOSE2313), E347G (BOSE2323; BOSE3085), P492Q (BOSE2120; BOSE2303), H493Y (BOSE2538; BOSE3087), and T617A (BOSE2123; BOSE2307). B. Missense mutations that suppress the *spoIIIEΔγ* phenotype do not affect the function of full-length SpoIIIE. Site-directed mutagenesis was used to introduce each indicated mutation into a *spoIIIE* allele that was then expressed under its native promoter at *ycgO* in *ΔspoIIIE* strains. Each mutant sporulated as well as cells with wild-type *spoIIIE* (bKM776). Seven mutants were tested: P260L (BOSE2298), E312A (BOSE2290), Y316D (BOSE2296), E347G (BOSE2325), P492Q (BOSE2294), D586N (BOSE3091), and T617A (BOSE2292). All eight sporulation efficiencies were not significantly different from each other by single factor ANOVA (p = 0.503).

We tested whether suppressor mutants of *spoIIIEΔγ* were dominant or recessive to *spoIIIEΔγ* in strains with the suppressor allele at one ectopic locus and *spoIIIEΔγ* at another (Fig 5A). Each copy of *spoIIIEΔγ* was expressed from the native promoter. Like previously published ectopic *spoIIIE* expression constructs that fully complement null mutations in *spoIIIE*, each of these constructs includes 408 bp upstream of the *spoIIIE* start codon [12, 38]. For four of ten tested variants, a strain with both alleles sporulated less well than a strain that only harbors the *spoIIIEΔγ* suppressor allele. This phenotype is consistent with the formation of mixed oligomers containing both variants of SpoIIIEΔγ. Mutations that were dominant were not clustered in a specific domain or region of SpoIIIEΔγ, nor were they the weakest suppressors of the sporulation defect. Differences in the extents of dominance for the suppressor mutants may reflect varying propensity of mutants to form mixed oligomers with SpoIIIEΔγ.

Mutations that improve the function of SpoIIIEΔγ might improve or impair full-length SpoIIIE. We introduced seven of the suppressor mutations into full-length *spoIIIE*, and found that they did not significantly alter sporulation by exhaustion (Fig 5B).

### Conservation of residues at mutated sites

The identities of intragenic suppressor mutations and the positions at which they were found did not reveal any obvious trends. For some positions, more than one possible change could rescue sporulation. We examined how conserved these positions are in other SpoIIIE/FtsK family members. Because the linker domain is highly variable between family members, only mutations in the motor domain were considered. Interestingly, in only one instance (out of 874 total) was the residue corresponding to a suppressor mutation found in another family member. This is rather surprising given that the suppressor mutations did not have an adverse effect on sporulation in the context of full-length SpoIIIE. We found that the mutated positions exhibited varying extents of sequence conservation (Table D in S1 Text). We observed no significant differences between family members annotated as having or not having a γ domain.

### Mutants fail to rescue chromosome translocation defects during vegetative growth

**Fig 6:**
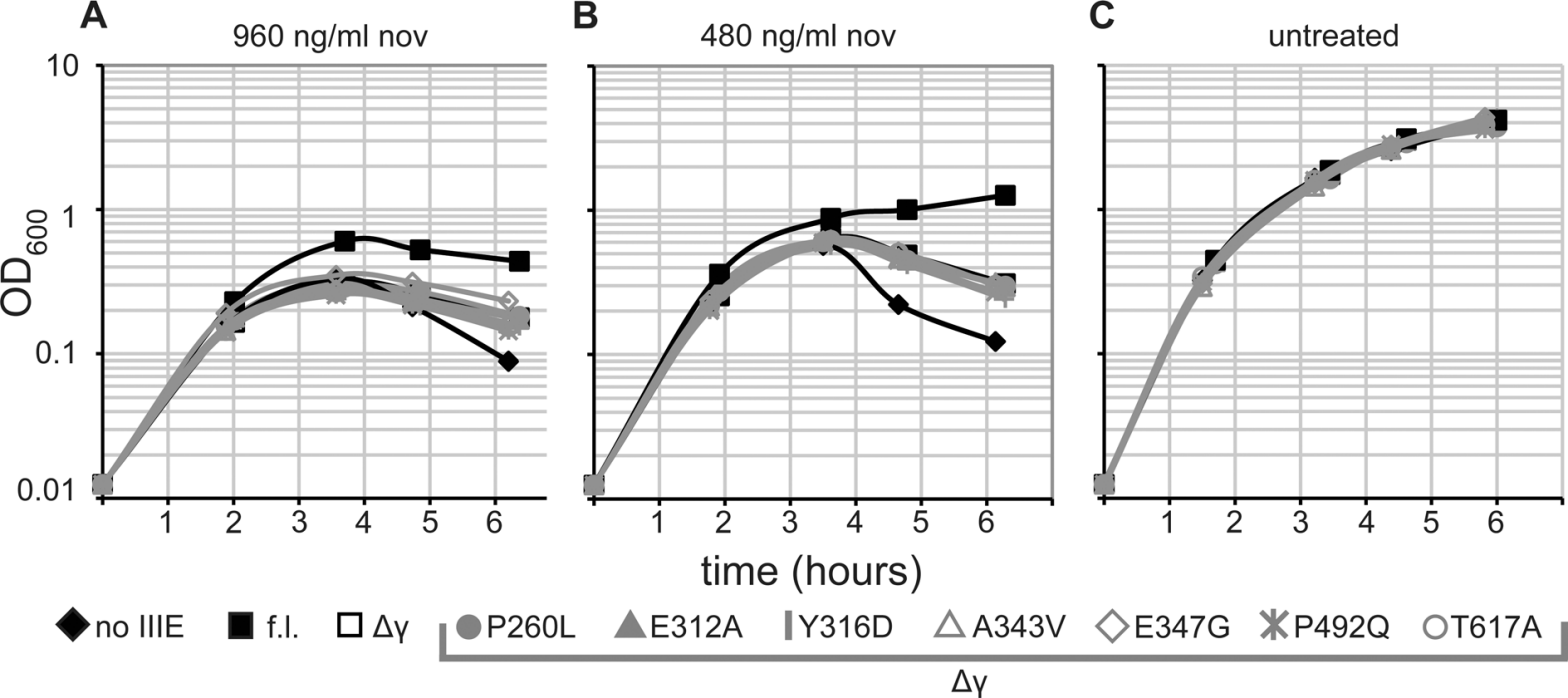
Mutations that rescue the sporulation defect of *spoIIIEΔγ* strains do not rescue a vegetative translocation defect. Growth of strains in various concentrations of the replication-stress-inducing antibiotic novobiocin (nov) was evaluated. Cells were grown to mid-exponential phase in LB, and then diluted to OD_600_ 0.0125 in LB containing 960 ng ml^-1^ nov (A), 480 ng ml^-1^ (B), or no nov (C). OD600 measurements are plotted versus hours after dilution. All strains harbor Δ*spoIIIE*::*neo*. As indicated, samples were from strains with no ectopic *spoIIIE* (bDR1066), *spoIIIE* (bKM776), *spoIIIEΔγ* (BOSE2042), or a *spoIIIEΔγ* mutant: P260L (BOSE2286), E312A (BOSE2121), Y316D (BOSE2284), A343V (BOSE2288), E347G (BOSE2323), P492Q (BOSE2120), T617A (BOSE2123). Representative data from one of at least two replicates are plotted.

We found that although the suppressor mutants rescue sporulation of the *spoIIIEΔγ* strain, they did not rescue SpoIIIE function during vegetative growth. SpoIIIE is only required for vegetative growth of *B*. *subtilis* when replication or chromosome partitioning is perturbed [46, 47]. A *ΔspoIIIE* mutant is more sensitive than wild-type *B*. *subtilis* to antibiotics, such as novobiocin, that inhibit DNA gyrase and cause replication stress [46]. We assayed growth of *B*. *subtilis* strains harboring different *spoIIIE* alleles in LB plus various concentrations of novobiocin (Fig 6). At 0.96 ug ml^-1^ novobiocin, a strain with wild-type *spoIIIE* reached a higher OD600 and exhibited less lysis than the *ΔspoIIIE* strain (Fig 6A). At 0.48 ug ml^-1^ novobiocin, a strain with wild-type *spoIIIE* exhibited moderately impaired growth, whereas a *ΔspoIIIE* strain showed appreciable lysis (Fig 6B). At both novobiocin concentrations, the phenotype of a *spoIIIEΔγ* strain was intermediate between that of wild-type and *ΔspoIIIE*, and six of six tested suppressor mutants behaved similarly to the *spoIIIEΔγ* strain (Fig 6A and 6B). All strains grew comparably to each other in the absence of novobiocin (Fig 6C).

This finding is significant because it reveals a role for the γ domain that cannot be rescued by the suppressor mutants. During vegetative growth in *E*. *coli*, the SpoIIIE homolog FtsK stimulates resolution of chromosome dimers by XerC & XerD recombinases via direct contacts between the FtsK γ domain and XerD [28, 48]. However, SpoIIIE is dispensable for the corresponding process in *B*. *subtilis* [49]. Thus, the SpoIIIE γ domain is likely to be important for survival of replication stress during vegetative growth for other reasons. The roles of SpoIIIE during vegetative growth and during sporulation may differ due to the way the chromosome is organized in each state. Or SpoIIIE may interact, in a γ-domain-dependent manner, with different cell division components during sporulation and vegetative growth.

## Discussion

We set out to learn more about the role of the SpoIIIE γ domain in sporulating cells by exploring how cells would cope with its absence. We selected suppressors of the sporulation defect in *spoIIIEΔγ* strains by successive rounds of sporulation and outgrowth and found that spontaneously arising intragenic mutations could rescue sporulation to near-wild-type levels. The finding that a missense mutation can somehow compensate for the loss of a domain that senses DNA sequences and directs transport is surprising. The intragenic suppressors are all single missense mutations found throughout the linker and motor domains of SpoIIIE. They dramatically improve chromosome transport. Rescue does not require the SpoIIIE homolog SftA. We found that the intragenic suppressors have the same *in vivo* protein levels and *in vitro* ATPase activity as SpoIIIEΔγ, and the mechanism by which sporulation and chromosome transport are significantly improved remains unclear. The suppressor mutants vary in their effects when co-expressed with wild-type *spoIIIEΔγ*, perhaps indicating varying propensities to form mixed oligomers. The corresponding mutations in full-length *spoIIIE* do not alter sporulation levels. While the suppressors dramatically rescue sporulation, they do not rescue chromosome transport during vegetative growth, indicating that under certain conditions the function of the γ domain cannot be substituted for by these missense mutations.

Strikingly, we isolated many different mutations within *spoIIIEΔγ* that improve spore formation. The mechanism by which they rescue sporulation and chromosome transport *in vivo* has yet to be determined. We hypothesized that they might transport DNA more quickly. In this case, we would expect that the ATPase rate of the mutants would be higher than that of SpoIIIEΔγ, especially in the presence of genomic DNA. However, we found that the ATPase rate was unchanged in the mutants, even in the presence of *B*. *subtilis* DNA. Given the unaltered phenotype of the SpoIIIEΔγ suppressor mutants in these assays, we conclude that the mutants have not recovered the ability to detect specific DNA sequences nor are they likely to have acquired the ability to translocate DNA faster. However, it is possible that the mutant translocases work differently *in vivo*, and that this effect is not recapitulated *in vitro*.

We propose two alternative models to explain rescue: directional commitment and development delay. In the directional commitment model, suppressor mutations make the protein more likely to commit to transport DNA in one direction, rather than frequently pausing and reversing direction as was previously observed for *spoIIIEΔγ* cells. If each SpoIIIE complex committed absolutely to transport in one arbitrarily-determined direction, we might expect up to 75% of the cells to sporulate, leading to near-wild-type levels in the sporulation assay. This assumes there is one complex per arm of the chromosome, and that at least one must initiate DNA translocation in the right direction for successful sporulation. This minor defect would give near wild-type levels in the sporulation assay, so our data are consistent with this model.

Our findings that the suppressors do not rescue the requirement for chromosome segregation during vegetative growth are also consistent with the directional commitment model, if we consider the spatial orientation of the chromosomes in each case. During sporulation the chromosomes are properly oriented, but during replication stress in vegetative growth, spatial positioning of the chromosomes should be perturbed. In the absence of proper partitioning and localization of the genomic DNA, many complexes of SpoIIIE may be involved in translocation across vegetative septa. If each complex arbitrarily chooses a direction for transport, we would expect mis-segregation and guillotining of the DNA. Thus according to the directional commitment model, the differences we observe between sporulation and vegetative growth are due to the arrangement of DNA under each condition rather than some aspect of the growth conditions such as specific components of cellular division machinery or nutritional availability.

Alternatively, in the development delay model the suppressors would rescue sporulation by delaying the progression of spore formation. SpoIIIE is required for membrane fission (separation of the mother cell and forespore membranes) and involved in membrane fusion (joining of mother cell membranes that have migrated around the forespore to complete engulfment)[12, 14, 18, 19, 20]. If the suppressor mutations slow down these processes, it is possible that they would allow the cells enough extra time to successfully complete DNA transport and develop viable spores. According to this model, the lack of rescue we observe during vegetative growth would be due to the fact that very different mechanisms and factors are involved in sporulation and vegetative division. The mechanism by which progression of sporulation is delayed is unlikely to delay vegetative cell division.

Importantly, the apparent increase in successful transport of the terminus into the forespore (Fig 2B) does not distinguish between the two models proposed here. This observation could result from faster chromosome transport or from stabilizing cells that would otherwise lyse due to failed chromosome transport. Future experiments utilizing time-lapse microscopy should help to distinguish between the two models. Faster chromosome translocation in each cell would support the directional commitment model, whereas lengthening of the time between asymmetric septation and completion of engulfment would support the developmental delay model.

Thus, the intragenic suppressors of *spoIIIEΔγ* that we isolated and characterized in this study have revealed new questions about the mechanism by which SpoIIIE functions and about the roles SpoIIIE plays during sporulation and vegetative growth. If further studies support the directional commitment model, these SpoIIIEΔγ mutants would be valuable tools for mechanistic analysis. Understanding how the protein could become “locked” into pumping in one direction would provide insight into how wild-type SpoIIIE pumps DNA. If further studies support the developmental delay model, perhaps the search for proteins that interact with SpoIIIE would be aided by using the suppressor mutants.

## Supporting Information

S1 Fig Schematic of *spoIIIE* sequences. *spoIIIE* and nearby orfs are shown as green arrows. The extents of deleted sequences in three *ΔspoIIIE* alleles are indicated with pink bars. The sequence present in ectopic *spoIIIEΔγ* constructs at *ycgO* and *yhdGH* is indicated by the yellow bar. The extent of sequences on either side of the deletion-insertions that are identical to sequences present in ectopic *spoIIIEΔγ* constructs is as follows: 672 bp and 145 bp for *ΔspoIIIE*::*spc*, 525 bp and 179 bp for *ΔspoIIIE*::*neo*, and 201 bp and none for *ΔspoIIIE*::*mls*. The *ΔspoIIIE*::*mls* allele truncates the *ylzJ* gene.
(TIFF)

S2 Fig Intragenic suppressor mutations do not alter protein levels. SpoIIIE levels are similar in strains expressing *spoIIIEΔγ* or a suppressor allele. Western blots of all three replicate sets of samples are shown here. Uncropped images are shown, except in panel A, where the left portion of the blot was cropped out, because it showed unrelated samples. One cropped image representative of these three blots is shown in Fig 4A. Samples were harvested 2.5 h after cells were induced to sporulate by resuspension. Protein levels were evaluated using Western blots with antibodies against SpoIIIE. Arrows indicate the positions of full-length SpoIIIE and SpoIIIEΔγ on the blots. Asterisks indicate SpoIIIEΔγ variants whose migration was altered. All strains bear Δ*spoIIIE*::*neo*. As indicated, samples were from strains with full-length (“f.l.”) *spoIIIE* (1:10 or 1:100 dilution; bKM776), no ectopic *spoIIIE* (bDR1066), *spoIIIEΔγ* (BOSE2042), or a *spoIIIEΔγ* mutant: P260L (BOSE2286), S264I (BOSE2540), E310K (BOSE2411), E312A (BOSE2121), Y316D (BOSE2284), P319S (BOSE2321), A343V (BOSE2288), E347G (BOSE2323), P492Q (BOSE2120), H493Y (BOSE2538), T617A (BOSE2123). “Std 1” indicates Magic Mark XP Western Standards (Invitrogen). “Std 2” indicates Precision Plus Protein Dual Color Standards (Bio-Rad). A-B. Samples from replicate 1, except for the last four lanes in the blot shown in B, which are from replicate 2. C. Samples from replicate 2. D. Samples from replicate 3.
(TIF)

S1 Text This file contains supplemental materials and methods describing strain and plasmid construction and Tables A-D. Table A, *B*. *subtilis* strains used in this study. Table B, Primers used in this study. Table C, Suppressor selection summary. Table D, Residues in SpoIIIE/FtsK family members corresponding to mutated residues in SpoIIIEΔγ suppressors.
(PDF)
