# Supplementary figures and images for: Missense Mutations Allow a Sequence-Blind Mutant of SpoIIIE to Successfully Translocate Chromosomes during Sporulation
Source: PLoS One. 2016 Feb 5;11(2):e0148365. doi: 10.1371/journal.pone.0148365 (PMC4744071; doi:10.1371/journal.pone.0148365)

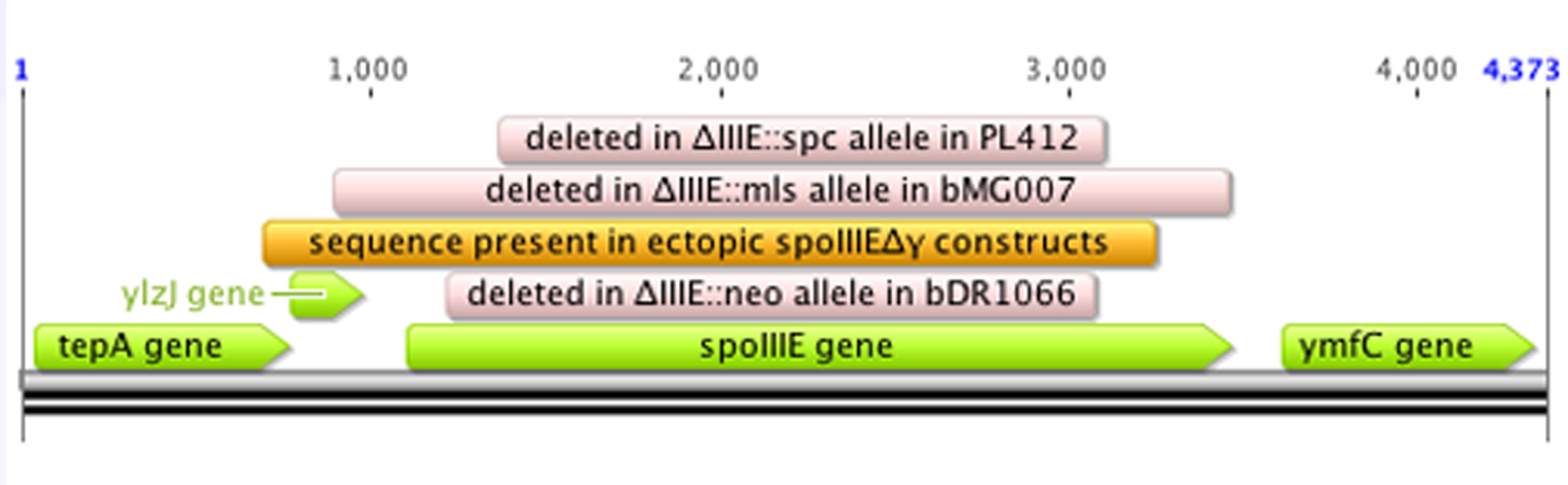

Supplement: S1 Fig — spoIIIE and nearby orfs are shown as green arrows. The extents of deleted sequences in three ΔspoIIIE alleles are indicated with pink bars. The sequence present in ectopic spoIIIEΔγ constructs at ycgO and yhdGH is indicated by the yellow bar. The extent of sequences on either side of the deletion-insertions that are identical to sequences present in ectopic spoIIIEΔγ constructs is as follows: 672 bp and 145 bp for ΔspoIIIE::spc, 525 bp and 179 bp for ΔspoIIIE::neo, and 201 bp and none for ΔspoIIIE::mls. The ΔspoIIIE::mls allele truncates the ylzJ gene. (TIFF) [file pone.0148365.s001.tiff]

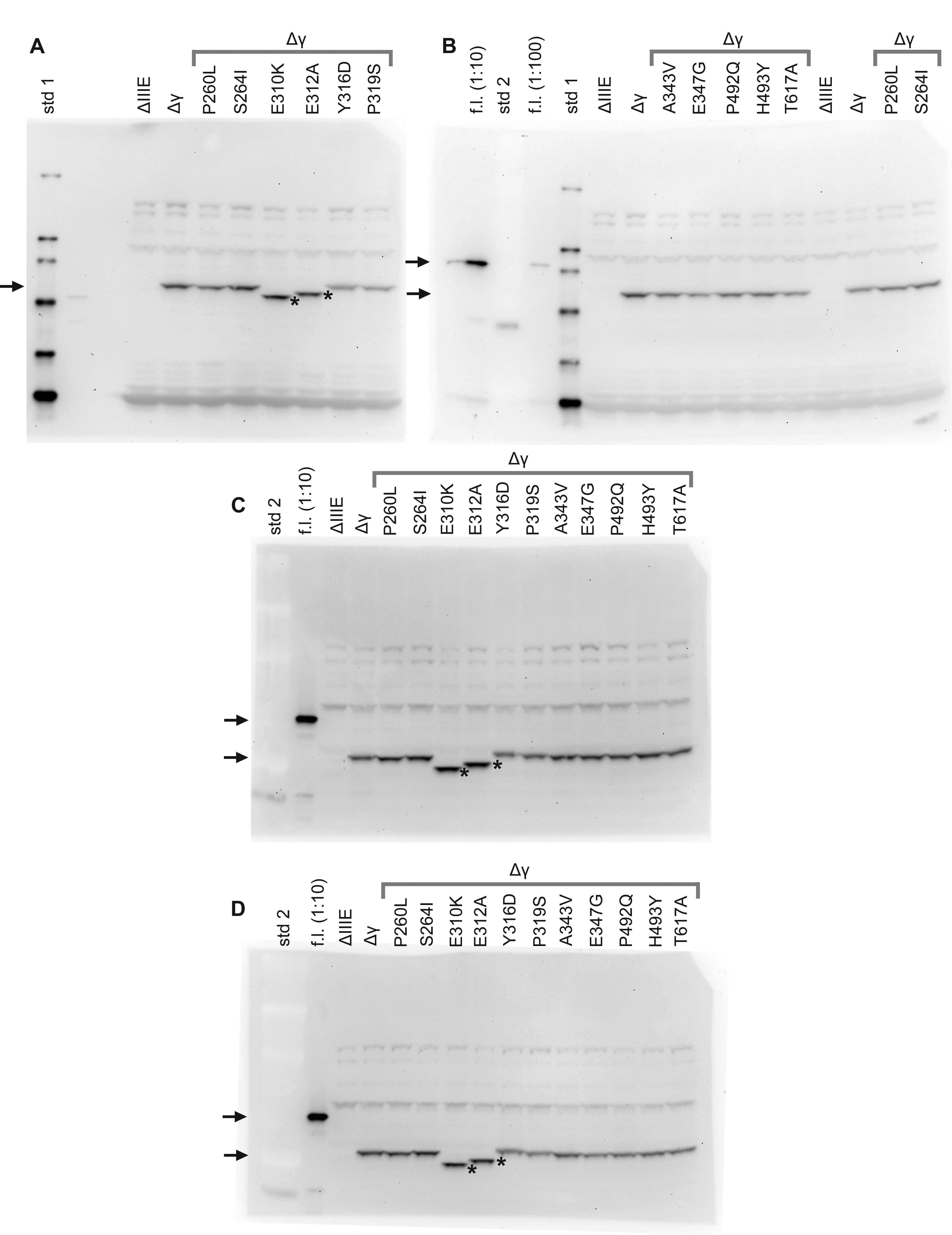

Supplement: S2 Fig — SpoIIIE levels are similar in strains expressing spoIIIEΔγ or a suppressor allele. Western blots of all three replicate sets of samples are shown here. Uncropped images are shown, except in panel A, where the left portion of the blot was cropped out, because it showed unrelated samples. One cropped image representative of these three blots is shown in Fig 4A. Samples were harvested 2.5 h after cells were induced to sporulate by resuspension. Protein levels were evaluated using Western blots with antibodies against SpoIIIE. Arrows indicate the positions of full-length SpoIIIE and SpoIIIEΔγ on the blots. Asterisks indicate SpoIIIEΔγ variants whose migration was altered. All strains bear ΔspoIIIE::neo. As indicated, samples were from strains with full-length (“f.l.”) spoIIIE (1:10 or 1:100 dilution; bKM776), no ectopic spoIIIE (bDR1066), spoIIIEΔγ (BOSE2042), or a spoIIIEΔγ mutant: P260L (BOSE2286), S264I (BOSE2540), E310K (BOSE2411), E312A (BOSE2121), Y316D (BOSE2284), P319S (BOSE2321), A343V (BOSE2288), E347G (BOSE2323), P492Q (BOSE2120), H493Y (BOSE2538), T617A (BOSE2123). “Std 1” indicates Magic Mark XP Western Standards (Invitrogen). “Std 2” indicates Precision Plus Protein Dual Color Standards (Bio-Rad). A-B. Samples from replicate 1, except for the last four lanes in the blot shown in B, which are from replicate 2. C. Samples from replicate 2. D. Samples from replicate 3. (TIF) [file pone.0148365.s002.tif]
