# Supplementary material for: Missense Mutations Allow a Sequence-Blind Mutant of SpoIIIE to Successfully Translocate Chromosomes during Sporulation
Source: PLoS One. 2016 Feb 5;11(2):e0148365. doi: 10.1371/journal.pone.0148365 (PMC4744071; doi:10.1371/journal.pone.0148365)
Supplement: S1 Text — Table A, B. subtilis strains used in this study. Table B, Primers used in this study. Table C, Suppressor selection summary. Table D, Residues in SpoIIIE/FtsK family members corresponding to mutated residues in SpoIIIEΔγ suppressors. (PDF) [file pone.0148365.s003.pdf]

Missense Mutations Allow a Sequence-blind Mutant of SpoIIIE to Successfully Translocate Chromosomes During Sporulation

SUPPLEMENTAL MATERIALS AND METHODS

Strain and plasmid construction

B. subtilis strains used in this study are listed in Table A in S1 text and are all derivatives of the prototrophic strain PY79, unless otherwise noted [1]. Alleles were introduced to *B. subtilis* strains by transformation. Fresh streaks were used to inoculate MC medium [2], cells were grown for 4 hours at 37°C, DNA was added, and cells were grown for an additional 1 to 2 hours before plating. The *E. coli* strain DH5α was used for all cloning and for purification of soluble SpoIIIE variants. Primers used to generate new plasmids for this study are listed in Table B in S1 text. All site-directed mutagenesis was performed by Quikchange (Stratagene). Relevant portions of all generated plasmids were sequenced (Genewiz).

Because of gene conversions that restored *spoIIIE* at the native locus obtained during suppressor selection, for any strain exhibiting near wild-type sporulation efficiency, the presence of the *ΔspoIIIE* allele was confirmed by PCR.

The *ycgO::{spoIIIEΔγ cat}* allele was previously used, but not described [3]. It was generated by transformation of bDR2036 (*PY79 ycgO::spc*; D.Z. Rudner, HMS) with pBB273; double crossovers were detected by marker replacement of *spc*. To construct pBB273, *spoIIIEΔγ* was amplified from PY79 genomic DNA using primers oBMB162 and oBMB242. The resulting fragment was ligated into pKM063 (double-crossover vector for

integration with chloramphenicol resistance at *ycgO*; K.A. Marquis & D.Z. Rudner, HMS) by EcoRI/BamHI.

Plasmids to integrate *spoIIIEΔγ* mutants at *ycgO* with *cat* were generated by mutagenesis of pBB273. Plasmids for integration of *spoIIIE* at *ycgO* with *cat* were generated by mutagenesis of pBB078 [4]. Plasmids for integration of alleles at *ycgO* were used to transform bDR2036, and double-crossovers were detected by marker replacement of *spc*.

The *yhdGH::{spoIIIEΔγ mls}* allele was generated by transformation of bBB445 (*PY79 yhdGH::cat*) with pBOSE2203; double-crossovers were detected by marker replacement of *cat*. To construct pBOSE2203, a fragment including *spoIIIEΔγ* was amplified from bBB388 genomic DNA using oBMB162 and oBMB242, and the fragment was ligated into pBB279 (double-crossover vector for integration with mls-resistance at *yhdGH;* [5]) by EcoRI/BamHI. The promoter and coding sequences for *spoIIIEΔγ* in this construct are identical to those in pBB273, used to make the *ycgO::{spoIIIEΔγ cat}* allele.

Several new strains were generated for *spoIIIEΔγ* suppressor selection. The codon for P492 in *spoIIIEΔγ* was mutated from ccg to cct by mutagenesis of pBB273 and the resulting plasmid was used to create BOSE2254. BOSE2254 was then transformed with *ΔspoIIIE::neo* from bDR1066 to generate BOSE2282, *ΔspoIIIE::spc* from PL412 [6] to generate BOSE2425, and *ΔspoIIIE::mls* from bMG007 to generate BOSE2427. The extents of the *ΔspoIIIE::neo* and *ΔspoIIIE::spc* deletions (S1 Fig) were determined by PCR of bDR1066 and PL412 genomic DNA and sequencing of the PCR products.

bDR1066 (D.Z. Rudner, HMS) was made by crossing a partial replacement of *spoIIIE* with *aphA-3* [7] into PY79 twice.

bMG007 was made by transforming PY79 with pMG008, a plasmid for integration of a mls-resistance marker by double crossover at *spoIIIE*. The double crossover was verified by PCR. pMG008 was generated by inserting upstream (oBMB284+oBMB285; EagI/SalI) and downstream (oBMB286+oBMB287; EcoRI/XbaI) *spoIIIE*-flanking sequences from PY79 into pKM074 [5] on either side of *cat*. The *cat* gene was then replaced with an mls-resistance gene from pKM084 (K.A. Marquis & D.Z. Rudner, HMS) using BamHI/SalI.

Strains for the cfp/yfp microscopy assay were made by transforming bBB115 (*ycgO::tet ΔspoIIIE::spc yycR::{PspoIIQ-yfp phleo} pelB::{PspoIIQ-cfp kan}*) with the indicated *ycgO* allele.

The *ΔsftA::spc* allele originated from SB1150, which we obtained from the BGSC [8]. This allele was crossed into PY79 twice to generate BOSE2074, and genomic DNA from the resulting strain was used for subsequent strain construction.

Plasmids for purification of soluble, His-tagged versions of SpoIIIEΔγ from *E. coli* were made by mutagenesis of pMB041. pMB041 was previously used to purify a soluble form of SpoIIIEΔγ but was not described [3]. pMB041 was made by using divergent primers (oMB165, oMB166) to PCR from template pJB103 [9], ligating together the resulting fragments, and transforming DH5α *E. coli* with the products.

References

1. Youngman P, Perkins JB, Losick R. Construction of a cloning site near one end of Tn917 into which foreign DNA may be inserted without affecting transposition in Bacillus subtilis or expression of the transposon-borne erm gene. Plasmid. 1984;12(1):1-9. PubMed PMID: 6093169.
2. Kunst F, Msadek T, Rapoport G. Signal-Transduction Network Controlling Degradative Enzyme-Synthesis and Competence in Bacillus-Subtilis. In: Piggot P, Moran C, Youngman P, editors. Regulation of Bacterial Differentiation. Washington, D.C.: American Society for Microbiology; 1994. p. 1-20.
3. Besprozvannaya M, Pivorunas VL, Feldman Z, Burton BM. SpoIIIE protein achieves directional DNA translocation through allosteric regulation of ATPase activity by an accessory domain. The Journal of biological chemistry. 2013;288(40):28962-74. doi: 10.1074/jbc.M113.484055. PubMed PMID: 23974211; PubMed Central PMCID: PMC3789994.
4. Burton BM, Marquis KA, Sullivan NL, Rapoport TA, Rudner DZ. The ATPase SpoIIIE transports DNA across fused septal membranes during sporulation in Bacillus subtilis. Cell. 2007;131(7):1301-12. Epub 2007/12/28. doi: S0092-8674(07)01413-4 [pii] 10.1016/j.cell.2007.11.009. PubMed PMID: 18160039; PubMed Central PMCID: PMC2913279.
5. Bernard R, Marquis KA, Rudner DZ. Nucleoid occlusion prevents cell division during replication fork arrest in Bacillus subtilis. Mol Microbiol. 2010;78(4):866-82. doi: 10.1111/j.1365-2958.2010.07369.x. PubMed PMID: 20807205; PubMed Central PMCID: PMC2978284.
6. Pogliano K, Hofmeister AE, Losick R. Disappearance of the sigma E transcription factor from the forespore and the SpoIIE phosphatase from the mother cell contributes to establishment of cell-specific gene expression during sporulation in Bacillus subtilis. J Bacteriol. 1997;179(10):3331-41. Epub 1997/05/01. PubMed PMID: 9150232; PubMed Central PMCID: PMC179115.
7. Wu LJ, Errington J. Bacillus subtilis SpoIIIE protein required for DNA segregation during asymmetric cell division. Science. 1994;264(5158):572-5. Epub 1994/04/22. PubMed PMID: 8160014.
8. Biller SJ, Burkholder WF. The Bacillus subtilis SftA (YtpS) and SpoIIIE DNA translocases play distinct roles in growing cells to ensure faithful chromosome partitioning. Mol Microbiol. 2009;74(4):790-809. doi: 10.1111/j.1365-2958.2009.06893.x. PubMed PMID: 19788545.
9. Bath J, Wu LJ, Errington J, Wang JC. Role of Bacillus subtilis SpoIIIE in DNA transport across the mother cell-prespore division septum. Science. 2000;290(5493):995-7. Epub 2000/11/04. doi: 8957 [pii]. PubMed PMID: 11062134.

SUPPLEMENTAL TABLES

Table A. *B. subtilis* strains used in this study.

Strain	Genotype[a,b]	Source/reference
PY79	Prototrophic derivative of *B. subtilis* 168	[1]
bBB128	*ycgO::{spoIIIE cat} ΔspoIIIE::spc yycR::{PspoIIQ-yfp phleo} pelB::{PspoIIQ-cfp kan}*	[4]
bBB388	*ycgO::{spoIIIEΔγ cat} ΔspoIIIE::spc*	This study
bBB412	*ycgO::{spoIIIEΔγ cat} ΔspoIIIE::spc yycR::{PspoIIQ-yfp phleo} pelB::{PspoIIQ-cfp kan}*	This study
bDR1066	*ΔspoIIIE::neo*	D.Z. Rudner, HMS
bKM776	*ycgO::{spoIIIE cat} ΔspoIIIE::neo*	[4]
BOSE2042	*ycgO::{spoIIIEΔγ cat} ΔspoIIIE::neo*	This study
BOSE2120	*ycgO::{spoIIIEΔγ(P492Q) cat} ΔspoIIIE::neo*	This study
BOSE2121	*ycgO::{spoIIIEΔγ(E312A) cat} ΔspoIIIE::neo*	This study
BOSE2123	*ycgO::{spoIIIEΔγ(T617A) cat} ΔspoIIIE::neo*	This study
BOSE2200	*ycgO::{spoIIIEΔγ(P492Q) cat} ΔspoIIIE::spc yycR::{PspoIIQ-yfp phleo} pelB::{PspoIIQ-cfp kan}*	This study
BOSE2201	*ycgO::{spoIIIEΔγ(E312A) cat} ΔspoIIIE::spc yycR::{PspoIIQ-yfp phleo} pelB::{PspoIIQ-cfp kan}*	This study
BOSE2202	*ycgO::{spoIIIEΔγ(T617A) cat} ΔspoIIIE::spc yycR::{PspoIIQ-yfp phleo} pelB::{PspoIIQ-cfp kan}*	This study
BOSE2282	*ycgO::{spoIIIEΔγ [P492(ccg--> cct)] cat} ΔspoIIIE::neo*	This study
BOSE2284	*ycgO::{spoIIIEΔγ(Y316D) cat} ΔspoIIIE::neo*	This study
BOSE2286	*ycgO::{spoIIIEΔγ(P260L) cat} ΔspoIIIE::neo*	This study
BOSE2288	*ycgO::{spoIIIEΔγ(A343V) cat} ΔspoIIIE::neo*	This study
BOSE2290	*ycgO::{spoIIIE(E312A) cat} ΔspoIIIE::neo*	This study
BOSE2292	*ycgO::{spoIIIE(T617A) cat} ΔspoIIIE::neo*	This study

BOSE2294	*ycgO::{spoIIIE(P492Q) cat} ΔspoIIIE::neo*	This study
BOSE2296	*ycgO::{spoIIIE(Y316D) cat} ΔspoIIIE::neo*	This study
BOSE2298	*ycgO::{spoIIIE(P260L) cat} ΔspoIIIE::neo*	This study
BOSE2301	*yhdGH::{spoIIIEΔγ mls} ycgO::{spoIIIEΔγ cat} ΔspoIIIE::neo*	This study
BOSE2303	*yhdGH::{spoIIIEΔγ mls} ycgO::{spoIIIEΔγ(P492Q) cat} ΔspoIIIE::neo*	This study
BOSE2305	*yhdGH::{spoIIIEΔγ mls} ycgO::{spoIIIEΔγ(E312A) cat} ΔspoIIIE::neo*	This study
BOSE2307	*yhdGH::{spoIIIEΔγ mls} ycgO::{spoIIIEΔγ(T617A) cat} ΔspoIIIE::neo*	This study
BOSE2309	*yhdGH::{spoIIIEΔγ mls} ycgO::{spoIIIEΔγ(Y316D) cat} ΔspoIIIE::neo*	This study
BOSE2311	*yhdGH::{spoIIIEΔγ mls} ycgO::{spoIIIEΔγ(P260L) cat} ΔspoIIIE::neo*	This study
BOSE2313	*yhdGH::{spoIIIEΔγ mls} ycgO::{spoIIIEΔγ(A343V) cat} ΔspoIIIE::neo*	This study
BOSE2321	*ycgO::{spoIIIEΔγ(P319S) cat} ΔspoIIIE::neo*	This study
BOSE2323	*ycgO::{spoIIIEΔγ(E347G) cat} ΔspoIIIE::neo*	This study
BOSE2325	*ycgO::{spoIIIE(E347G) cat} ΔspoIIIE::neo*	This study
BOSE2331	*ycgO::{spoIIIEΔγ(P260L) cat} ΔspoIIIE::spc yycR::{PspoIIQ-yfp phleo} pelB::{PspoIIQ-cfp kan}*	This study
BOSE2332	*ycgO::{spoIIIEΔγ(A343V) cat} ΔspoIIIE::spc yycR::{PspoIIQ-yfp phleo} pelB::{PspoIIQ-cfp kan}*	This study
BOSE2425	*ycgO::{spoIIIEΔγ[P492(ccg--> cct)] cat} ΔspoIIIE::spc*	This study
BOSE2427	*ycgO::{spoIIIEΔγ[P492(ccg--> cct)] cat} ΔspoIIIE::mls*	This study
BOSE2486	*ycgO::{spoIIIEΔγ(E312A) cat} ΔspoIIIE::neo ΔsftA::spc*	This study
BOSE2488	*ycgO::{spoIIIEΔγ(T617A) cat} ΔspoIIIE::neo ΔsftA::spc*	This study
BOSE2490	*ycgO::{spoIIIEΔγ(Y316D) cat} ΔspoIIIE::neo ΔsftA::spc*	This study
BOSE2492	*ycgO::{spoIIIEΔγ(P260L) cat} ΔspoIIIE::neo ΔsftA::spc*	This study
BOSE2494	*ycgO::{spoIIIEΔγ(A343V) cat} ΔspoIIIE::neo ΔsftA::spc*	This study
BOSE2496	*ycgO::{spoIIIEΔγ(P319S) cat} ΔspoIIIE::neo ΔsftA::spc*	This study

BOSE2498	*ycgO::{spoIIIEΔγ(E347G) cat} ΔspoIIIE::neo ΔsftA::spc*	This study
BOSE2538	*ycgO::{spoIIIEΔγ(H493Y) cat} ΔspoIIIE::neo*	This study
BOSE2540	*ycgO::{spoIIIEΔγ(S264I) cat} ΔspoIIIE::neo*	This study
BOSE2935	*ycgO::{spoIIIEΔγ(P492Q) cat} ΔspoIIIE::neo ΔsftA::spc*	This study
BOSE3083	*yhdGH::{spoIIIEΔγ mls} ycgO::{spoIIIEΔγ(P319S) cat} ΔspoIIIE::neo*	This study
BOSE3085	*yhdGH::{spoIIIEΔγ mls} ycgO::{spoIIIEΔγ(E347G) cat} ΔspoIIIE::neo*	This study
BOSE3087	*yhdGH::{spoIIIEΔγ mls} ycgO::{spoIIIEΔγ(H493Y) cat} ΔspoIIIE::neo*	This study
BOSE3089	*yhdGH::{spoIIIEΔγ mls} ycgO::{spoIIIEΔγ(S264I) cat} ΔspoIIIE::neo*	This study
BOSE3091	*ycgO::{spoIIIE(D586N) cat} ΔspoIIIE::neo*	This study

[a] All strains are PY79 derivatives

[b] None of the listed strains are original isolates from the suppressor selection. All indicated mutations in *spoIIIEΔγ* were made as indicated in Materials and Methods. For each, site-directed mutagenesis was performed using a plasmid that was cloned in *E. coli*, sequenced, and introduced into the *B. subtilis* chromosome by double crossover at the indicated ectopic site. The resulting strains were transformed or genomic DNA was purified from them and used to transform other strains.

Table B. Primers used in this study.

Primer	Sequence (5' to 3')	Purpose
oBMB162	GTCAGAATTCGTCGGACAGGCAATCAAT	pBB273, pBOSE2203
oBMB242	GAGGATCCTCGAGTTATTAGGAATGAGTTTCCGTCGTCTC CTCAG	pBB273, pBOSE2203
oBMB284	GCGCGGCCGTGTTGAAGCTGGTCTTGCCATAGC	pMG008
oBMB285	GCCGTCGACCTTCCGCTTCATTCCCTTTCATTT	pMG008
oBMB286	CGGGAATTCGCTCTCTTCTTAATGAAGG	pMG008
oBMB287	GGCTCTAGATCATCGGAATTTTGTCTATAC	pMG008
oBOSE639	CCTTTACAGAGCTCGCAAATAAGGATTACGAGATGC	E312A
oBOSE640	GCATCTCGTAATCCTTATTTGCGAGCTCTGTAAAGG	E312A
oBOSE641	CCATCTTATTATTGCGGCACAGCGGCCATCGG	T617A
oBOSE642	CCGATGGCCGCTGTGCCGCAATAATAAGATGG	T617A
oBOSE643	GTATTTTAATGCGGGCGAAACAGCATGAAGTGAAAATGA TG	P492Q
oBOSE644	CATCATTTTCACTTCATGCTGTTTCGCCCGCATTAAAATAC	P492Q
oBOSE645	GCTCGAAAATAAGGATGACGAGATGCCGTCACTGG	Y316D
oBOSE646	CCAGTGACGGCATCTCGTCATCCTTATTTTCGAGC	Y316D
oBOSE647	CGCCTCTTATTCATTCAGAGCTGATTATCTCAAGCTTTTCT G	P260L
oBOSE648	CAGAAAAGCTTGAGATAATCAGCTCTGAATGAATAAGAG GCG	P260L
oBOSE649	CGGCCAGCAGGCTGATAAAAAGAATATTTATGAAAATGT GAGAAAGCTTGAACGCACATTCC	A343V
oBOSE650	GGAATGTGCGTTCAAGCTTTCTCACATTTTCATAAATATTC TTTTTATCAGCCTGCTGGCCG	A343V
oBOSE651	GTATTTTAATGCGGGCGAAACCTCATGAAGTGAAAATGA TG	P492 (ccg to cct)
oBOSE652	CATCATTTTCACTTCATGAGGTTTCGCCCGCATTAAAATAC	P492 (ccg to cct)
oBOSE653	GGATTACGAGATGTCGTCACTGGATTTGCTGGC	P319S
oBOSE654	GCCAGCAAATCCAGTGACGACATCTCGTAATCC	P319S
oBOSE655	GCGAGAAAGCTTGGACGCACATTCCAAAGCTTTGG	E347G
oBOSE656	CCAAAGCTTTGGAATGTGCGTCCAAGCTTTCTCGC	E347G
oBOSE660	CTGCTCCTCCTATGACCTTTACAAAGCTCGAAAATAAGG	E310K
oBOSE661	CCTTATTTTCGAGCTTTGTAAAGGTCATAGGAGGAGCAG	E310K
oBOSE702	GCGGGCGAAACCGTATGAAGTGAAAATGATGATG	H493Y
oBOSE703	CATCATCATTTTCACTTCATACGGTTTCGCCCGC	H493Y
oBOSE704	GAGCCGATTATCTCAATCTTTTCTGATCGTAATGAAGAGG	S264I
oBOSE705	CCTCTTCATTACGATCAGAAAAGATTGAGATAATCGGCTC	S264I
oMB165	Phos-CAGGTCACCACCACCACCACCAC	pMB041
oMB166	Phos-GAGTTTCCGTCGTCTCCTCAGGAATCATTTCTTCTTG	pMB041

Table C. Suppressor selection summary

spoIIIEΔγ, ΔspoIIIE::spc[a]		
perp. cult.[e]	outcome[f]	rescue round[g]
1	Y316D	4
2	extragenic	19
3	E310K	21
4	P492Q	13
5	P492Q	18
6-1	extragenic	21
6-2	extragenic	21
7	extragenic	14
8	conversion	4
9	conversion	4
10	P319S	18
11	P492Q	11
12-1	E347G	18
12-2	extragenic	18
13	extragenic	14
14	no rescue	(>21)
15	P260L	9
16	A343V	5
17	extragenic	16
18	P492Q	4
19	A343V	13
20	extragenic	18
21	E312A	4
22	P492Q	4
23	T617A	4

spoIIIEΔγ(P492 ccg-->cct), ΔspoIIIE:neo[b]		
perp. cult.[e]	outcome[f]	rescue round[g]
1	Y316S	3
2	P319S	3
3	conversion	3
4	A343V	13
5	rearranged	8
6	A343V	13
7	unknown	42
8	conversion	4
9	A343V	4
10	conversion	10
11	unknown	35
12	conversion	8
13	conversion	10
14	Y316C	3
15	conversion	8
16	conversion	7
17	lost	5
18	conversion	13
19	A343V	3
20	extragenic	8

spoIIIEΔγ(P492 ccg-->cct), ΔspoIIIE:spc[c]		
perp. cult.[e]	outcome[f]	rescue round[g]
1	P319S	12
2	D586N	25
3	extragenic	25
4	E310K	6
5	E312K	15
6	H493Y	6
7	E312K	6
8	extragenic	25
9	E347G	15
10	S264I	15

spoIIIEΔγ(P492 ccg-->cct), ΔspoIIIE:mls[d]		
perp. cult.[e]	outcome[f]	rescue round[g]
1	A343V	12
2	E312K	15
3	S264I	28
4	unknown	15
5	A343V	12
6	Y316C	9
7	E310K	12
8	P319R	6
9	no rescue	(>28)
10	E312K	28

[a] abbreviated genotype of bBB388

[b] abbreviated genotype of BOSE2282

[c] abbreviated genotype of BOSE2425

[d] abbreviated genotype of BOSE2427

[e] perpetuated culture, term for one culture cycled repeatedly through sporulation, heat-kill, and germination

[f] for intragenic suppressors, mutation is specified; “extragenic”: suppressor isolate had wild-type *spoIIIEΔγ* sequence; “conversion”: denotes “gene conversion”, where *ΔspoIIIE* marker was lost and wild-type sporulation efficiency recovered; “no rescue”: perpetuated culture never exhibited increased sporulation efficiency; “rearranged”: rescue involved a rearrangement of sequences such that the strain harbored *ycgO::{ΔspoIIIEΔγ::neo cat} and spoIIIE[P492(ccg--> cct)]*; “unknown”: sequence of *spoIIIEΔγ* from isolates exhibiting rescue was not determined; “lost”: the perpetuated culture went missing during passaging

[g] approximate round of passaging during which culture exhibited rescue and individual isolates were recovered; for perpetuated cultures that never exhibited recovery (“no rescue”), the last round of passaging is indicated

Table D. Residues in SpoIIIE/FtsK family members corresponding to mutated residues in SpoIIIEΔγ suppressors[a]

	P319	A343	E347	P492	H493	D586	T617
A		39.3 (61.7)		17.9 (4.3)	6.0 (10.6)		
D					28.6 (14.9)	98.8 (92.9)	(4.3)
E			79.8 (80.9)		29.8 (63.8)		
G		2.4 (2.1)					
H				2.4			
I		1.2	2.4 (6.4)				
K		8.3 (12.8)	2.4		4.8 (4.3)		
L			6.0 (2.1)				
M			1.2				
N			(2.1)		7.1	1.2	
P	100 (85.1)			79.8 (89.4)			
Q			(4.3)		3.6 (2.1)		
R			(2.1)	(2.1)	14.3		
S		48.8 (21.3)	1.2		4.8		
T		(2.1)	1.2		1.2	(7.1)	100 (95.7)
V			6.0 (2.1)				
Y							
gaps	(14.9)			(4.3)	(4.3)		

[a]Percentages of different residues at each position in other family members, that have (no parentheses) or lack (parentheses) γ domains. Mutations found in suppressor isolates are highlighted with dark grey.
